# Preliminary analysis of lifestyle and genetic factors for hyperuricemia and gout prevalence in the Yunnan Miao population of China

**DOI:** 10.3389/fgene.2026.1729712

**Published:** 2026-01-29

**Authors:** Qiaohong Li, Salma Saeed Khan, Hao Yan, Weiying Kong, Lu Qin, Linmei Wu, Lingjie Li, Weijun Gong, Hua Zheng, Haiyan Li

**Affiliations:** 1 The First People’s Hospital of Yunnan Province, Kunming, China; 2 The People’s Hospital of Wuding County, Wuding, China; 3 Medical Faculty, Kunming University of Science and Technology, Kunming, China

**Keywords:** dietary and lifestyle factors, genetic predisposition, Miao ethnic group, SLC22A12, SLC2A9

## Abstract

**Objectives:**

Hyperuricemia and gout are common public health problems, stemming from both genetic and lifestyle factors. Evidence from multi-ethnic regions in Yunnan Province remains limited. This preliminary study examined hyperuricemia and gout prevalence, related biomarkers, lifestyle patterns, and *SLC2A9/SLC22A12* genetics variations among 88 participants from the Miao community in Yunnan Province China.

**Methods:**

A cross-sectional survey and biochemical study were conducted. Demographic and lifestyle data were collected, and blood samples were analyzed for serum biochemical indicators. Eight SNPs in *SLC2A9* and *SLC22A12* were genotyped. Logistic regression models were applied to allele and genotype data.

**Results:**

Demographic and clinical analyses for Miao villagers (n = 88) suggested that the morbidities of hyperuricemia and gout were more frequent in male and showed significant association with alcohol consumption, smoking, and elevated BMI. While dietary patterns showed no significant differences. Compared with non-hyperuricemia/non-gout individuals (n = 56), the hyperuricemia/gout group (n = 57) showed 56% higher uric acid (553.13 vs. 354.73 μmol/L), 37% elevated creatinine (84.66 vs. 61.80 μmol/L), and higher triglycerides (3.35 vs. 1.80 mmol/L), along with hematological abnormalities, e.g., elevated hemoglobin (162.77 vs. 147.50 g/L) and lower platelets counts (161.09 vs. 194.14 × 10^9^/L). Preliminary genetic analyses indicated a possible association between *SLC2A9_*rs10939650 and hyperuricemia/gout risk, whereas variant *SLC22A12* polymorphisms showed no association. After Bonferroni correction, no SNPs remained statistically significant.

**Conclusion:**

This preliminary study suggested that the relatively higher burden of hyperuricemia and gout in the Miao population may be influenced by ethnicity, sex, lifestyle factors, metabolic alteration, and potential genetic components. Given the small sample size, the genetic findings should be interpreted cautiously and validated in larger studies for that disease (hyperuricemia and gout) and for similar ethnic community.

## 1 Introduction

Gout, an inflammatory condition triggered by the accumulation of monosodium urate crystal in the joints, predominantly affects middle-aged and elderly individuals (Ahn, 2023). Hyperuricemia, the condition in which there is high concentration of uric acid in blood serum (serum uric acid ≥420 μmol/L), is a major risk factor for gout and is linked to diabetes, kidney disease, and cardiovascular/metabolic disorders (Du et al., 2024). Gout and hyperuricemia prevalence increased with genetic predisposition, unhealthy and poor diets, obesity, and metabolic syndrome (Kim et al., 2024; Zhang et al., 2022a), particularly in rural communities with distinct lifestyles (Dehlin et al., 2020). Generally, gout and hyperuricemia prevalence is unequal, with higher rates in developed countries due to dietary habits, lifestyle factors, poor nutrition (junk food), obesity, and diabetes (Kuo et al., 2015). In China, the burden of gout increases with age (peak at 55–64 years), primarily attributable to prolonged hyperuricemia and frequent comorbidities including hypertension, dyslipidemia, and chronic kidney disease (Xian et al., 2025; Xie et al., 2025). For example, Hypertriglyceridemia strongly linked with gout risk (He et al., 2024; Li et al., 2024a; Chang et al., 2025), while higher body mass index (BMI) exacerbates gout susceptibility in elderly men due to higher alcohol consumption and energy rich diets (Jin et al., 2024). For the Chinese population, two kidney-related genes were reported for influencing the incidence of gout, namely, the Low-Density Lipoprotein-Related Protein 2 (*LRP2*) and the Catechol-O-Methyltransferase (*COMT*) (Dong et al., 2015).

The Yunnan Province, home to multi-ethnic populations (Q. Zhang et al., 2021), is located in the southwestern border region of China (He et al., 2022). Statistically, although Yunnan exhibits a high hyperuricemia prevalence (15.8%), its gout prevalence is lower than that observed in Northeast China (24.6%) (Ying et al., 2019). These regional differences are attributed to variation in rural-urban dietary patterns, genetic background, alcohol consumption, and obesity (Huang et al., 2020). For example, traditional plant-based diets in the Southwest China region have been associated with lower rates of hyperuricemia and gout (Liu et al., 2018). The plant-based diets such as whole grains, vegetables, and fruits with low-purine properties play a significant role in lowering the risk of hyperuricemia and gout (Wu et al., 2023b). However, an increasing trend has emerged due to modern lifestyle factors, such as physical inactivity, high meat/seafood consumption, and frequent alcohol intake (Jakše et al., 2019; Zhu et al., 2022). A nationwide epidemiological survey (2015–2017) reported rising prevalence of hyperuricemia (17.7%) and gout (3.2%) compared with earlier national estimates (6.4% overall prevalence, with 7.9% in males and 4.9% in females) (Ji et al., 2025). Alarmingly, high uric acid levels are also becoming more common among Chinese children and adolescents, especially boys (26.6%), older teens (31.7%), and overweight individuals (50.6%) (Song et al., 2022). Overall, gout prevalence in China rises with age and is influenced by sexes, ethnicity, smoking, and metabolic diseases (Ji et al., 2025).

Genetic predispositions play a crucial role in hyperuricemia and gout. Many studies highlight the importance of two renal urate reabsorption transporters, such as GLUT9 and URAT1 (Pavelcova et al., 2020). GLUT9, encoded by *SLC2A9,* is a glucose transporter expressed in liver and kidney (DeBosch et al., 2014; Torres et al., 2014; Novikov et al., 2019; Chung and Kim, 2021). Variants such as rs16890979 in *SLC2A9* gene may impair urate reabsorption, leading to elevated serum uric acid level and promoting joint urate crystal formation (Torres et al., 2014). URAT1, encoded by the *SLC22A12* gene on chromosome 11, is an organic anion transporter (OAT) involved in renal urate handling (Stiburkova et al., 2013). Similarly, mutations in the *SLC22A12* gene can reduce uric acid excretion and contribute to hyperuricemia and gout (Perdomo-Ramírez et al., 2023). While *SLC2A9* and *SLC22A12* polymorphisms have been associated with serum uric acid levels in Asian populations, including Chinese, their significance varies by gender and genotype. For instance, the *SLC2A9*_rs3733591 variant was significantly associated with hyperuricemia only in females (*p* = 0.003), whereas *SLC22A12*_rs893006 showed no significant association in an elderly Chinese cohort (*p* = 0.061) (Liu J. et al., 2020). Despite these findings, the relevance of these genetic variants (*SLC2A9* and *SLC22A12)* to hyperuricemia and gout prevalence has not yet been studied in the Chinese Miao population.

Wuding county, located in north-central Yunnan, is a multi-ethnic region comprising 24 ethnic minority groups, including the Miao, Yi, and Hui peoples. Longyintan Village—a Miao community in Wuding county—exhibits hyperuricemia and gout prevalence. Although previous work reported that the prevalence of hyperuricemia is highest in the Miao people and lowest in the Hui people (Wu et al., 2017), no study has investigated the specific risk factors effecting the Miao population in Yunnan, China. Therefore, this cross-sectional study conducted in Longyintan Village (Yunnan, China) aimed to evaluate demographic, clinical, and genetic factors—including *SLC2A9* and *SLC22A12* variants—associated with hyperuricemia and gout prevalence among the Miao population.

## 2 Materials and methods

### 2.1 Subjects and sample collection

This study employed a household survey approach to investigate the prevalence of hyperuricemia and gout as well as associated with risk factors, among residents of Longyintan villagers. A total of 143 epidemiological questionnaires and 113 blood and urine samples were collected. As a Miao village, the Longyintan is a relatively isolated Miao village with a common pattern of internal marriages. In addition, 34 non-Miao residents from neighboring village were randomly selected as a control for the genetic comparisons, and blood samples were obtained from them. The questionnaire was designed according to the guidelines of the Chinese Society of Rheumatology (Tian et al., 2021).

Researchers, together with physicians from the Yunnan First People’s Hospital, Chuxiong Prefecture People’s Hospital and Maojie Town Health Center, conducted the questionnaire survey and collected biological samples. For each participant, demographic characteristics (including age, educational background), medical history (including family history of hyperuricemia/gout), and dietary habits were recorded. Besides, two tubes of approximately 3 mL of residual peripheral blood were collected from each participant, including both Miao residents of Longyintan and non-Miao individuals from neighboring villages, for biochemical analysis and genotyping. This study adhered to the principles of the declaration of Helsinki. Ethical approval was granted by the Ethics Committee of Yunnan First People’s Hospital and written informed consent was obtained from all participants.

### 2.2 Diagnostic criteria

According to the 2023 Multidisciplinary Expert Consensus on the Diagnosis and Treatment of Hyperuricemia Related Diseases in China, hyperuricemia is defined as a serum uric acid >420 μmol/L in both men and women (Li et al., 2024b). Gout was diagnosed based on the 2015 European Alliance of Associations for Rheumatology (EULAR)/American College of Rheumatology (ACR) classification criteria (Neogi et al., 2015).

### 2.3 Measurement of biochemical profiles

Following previously established protocols (Fischbach et al., 2021; Vargas-Morales et al., 2021), blood samples were analyzed for white blood cells (WBC), Red Blood Cell (RBC), Hemoglobin (Hb), Platelet Count (PLT), renal function (creatinine, and uric acid), and metabolic parameters including glucose, Total Cholesterol (TC), Triglycerides (TG), High-Density Lipoprotein Cholesterol (HDL-C), and Low-Density Lipoprotein Cholesterol (LDL-C). Urine samples underwent standardized urinalysis including pH, Specific gravity SG, nitrites, vitamin C, occult blood, bilirubin, urobilinogen, and leukocytes. All laboratory tests were performed at the Yunnan First People’s Hospital, under routine internal quality control procedures.

### 2.4 Genotyping of polymorphisms

Genomic DNA was extracted from EDTA-anticoagulated whole blood using a commercial kit according to the manufacturer’s instructions. Eight SNPs were analyzed: *SLC2A9* (rs16890979, rs10489070, rs2280205, rs3733591, rs10939650, and rs7442295) and *SLC22A12* (rs475688 and rs7929627). Genotyping was performed using the SNaPshot Multiplex Kit (Thermo Fisher Scientific) with primers designed using Primer3Plus (Varliero et al., 2021) (Table 1). PCR amplification was performed with initial denaturation at 95 °C for 2 min, followed by 11 cycles of 94 °C for 20 s, 65 °C for 40 s (decreasing by 0.5 °C per cycle), and 72 °C for 1.5 min, followed by 24 cycles of 94 °C for 20 s, 59 °C for 40 s and 72 °C for 1.5 min, and a final extension at 72 °C for 2 min. PCR products were purified with Exonuclease I/Shrimp Alkaline Phosphatase (ExoI/SAP; Thermo Fisher). SNaPshot extension and SAP cleanup (Promega) were performed before capillary electrophoresis using an ABI sequencer. Genotypes were analyzed with GeneMapper Software v6.5 (Liang et al., 2025).

**TABLE 1:** Details of Primers (Forward-F, Reverse-R) for Target Genes and its SNP.

SNP rsID	F-seq (5′-3′)	R-seq (5′-3′)
rs10489070	GAT​GAG​GTG​CTC​AAA​TGG​GAA​AG	AGG​TCT​AGC​ATG​GCC​CCA​GAT​T
rs10939650	CAG​ATA​GAG​ACA​GCC​TCC​TAC​TGA​CCT	GCT​CTG​GTC​TGT​GAC​TGT​GTC​CA
rs2280205	GAT​GCC​CCC​TGT​ACT​CAA​GGT​G	AAA​ATG​AGC​ATC​CAC​GCC​TCT​C
rs3733591-rs16890979	CAT​TGA​GGC​CAC​AGA​GCT​GGT​A	CCC​TGC​TGA​AAG​TCC​ATG​TGT​G
rs475688	ATG​GCA​GCT​TGA​CTC​CCA​ACA​C	GCC​CTG​CAT​CAG​CAT​GAG​AAC
rs7442295	GGT​GGC​TGG​GGC​TTA​AAA​TCA​C	AAG​AGG​GCT​GTC​CCC​AAA​GGT
rs7929627	CAC​TCA​GGA​CAG​GAT​ACC​CAG​ATG	GCC​CCC​ATT​TCT​GTG​GGT​AGA​G

rs3733591 and rs16890979 are located within the same PCR, amplicon; therefore, the same primer pair was used for both SNPs.

### 2.5 Statistical analysis

Statistical analysis was carried out using SPSS 26.0 (IBM Corp., Armonk, NY, United States) (Chen-Xu et al., 2019). Continuous variables were assessed for normality using the Shapiro-Wilk test. Normally distributed data (age, BMI, and uric acid) were presented as mean ± standard deviation (SD), whereas non-normally distributed data (e.g., WBC and creatinine) were summarized as median (interquartile range). Categorical variables were expressed as frequencies and percentages. Group comparisons between gout/hyperuricemia and non-gout/non-hyperuricemia groups were performed using independent t-tests for normally distributed variables and Mann-Whitney *U* tests (reported with *Z*-values) for skewed data. Chi-square or Fisher’s exact tests were used for categorical variables such as lifestyle factors, and dietary factors).

For genetic associations testing, multivariable logistic regression was performed to estimate unadjusted odds ratios (ORs) and 95% confidence intervals (CIs) under codominant, dominant, and recessive models. Covariates included age, sex, BMI, and ancestry principal components minimize the population stratification bias (Yang et al., 2014; Tseng et al., 2024). To account for multiple testing in genetic analyses, Bonferroni correction was applied for multiple comparisons (significance threshold = 0.05/number of SNPs) (Fan et al., 2024). Statistical significance was defined as *p* < 0.05 (two-tailed), with *p* < 0.01 considered highly significant (indicated by asterisks in tables). Hardy-Weinberg equilibrium (HWE) was tested for all SNPs to ensure genotyping quality (Yan et al., 2018; Cho et al., 2024). Linkage disequilibrium (LD) analysis was conducted using Haploview v4.2. Pairwise LD was calculated using the *r* ^2^ metric, and LD blocks were defined according to the Gabriel algorithm (Gabriel et al., 2002) with default confidence interval settings. SNPs with a minor allele frequency ≥0.05 and genotype call rate ≥95% were included, using the study population as the reference (Dong et al., 2015; Barrett et al., 2005). Besides, LD between SNPs was assessed using *R* ^2^ values (Chen et al., 2022).

## 3 Results

### 3.1 Demographic and clinical characteristics of participants

Although 143 villagers from the Longyintan Village (Total population: 224) participated in the initial screening, only 88 participants had complete questionnaires and biochemical information and were therefore included in the epidemiological analysis. Among them 39 participants were classified into non-hyperuricemia/non-gout group, and 49 into hyperuricemia/gout group (36 hyperuricemia, 13 gouts).

Significant demographic and clinical differences between two groups were observed (Table 2). Hyperuricemia and gout were substantially more common in men (83.7% vs. 28.2%) and were associated with a higher prevalence of pre-existing medical conditions (39.6% vs. 10.3%), %), non-traumatic joint pain (28.6% vs. 5.1%), and visible urate crystal deposits called tophi (10.6% vs. 0%). Lifestyle factors differed as well: frequent alcohol consumption (24.5% vs. 13.2%) and smoking (59.2% vs. 23.1%) were notably more common among affected individuals.

**TABLE 2:** Demographic and clinical comparisons between non-gout/non-hyperuricemia and gout/hyperuricemia groups.

Category	Subgroup	Hyperuricemia/Gout group (n = 49)	Non-Hyperuricemia/Non-gout group (n = 39)	χ^2^/t value	*p* value
Gender	Female	8 (16.33)	28 (71.79)	27.64	<0.001^*^
	Male	41 (83.67)	11 (28.21)	—	—
Family history of hyperuricemia	No	39 (79.59)	36 (92.31)	2.79	0.09
	Yes	10 (20.41)	3 (7.69)	—	—
Family history of gout	No	39 (79.59)	35 (89.74)	1.67	0.20
	Yes	10 (20.41)	4 (10.26)	—	—
Medical history	None	29 (60.42)	35 (89.74)	9.52	<0.001*
	Present	19 (39.58)	4 (10.26)	—	—
Non—traumatic joint pain	No	35 (71.43)	37 (94.87)	8.02	<0.001*
	Yes	14 (28.57)	2 (5.13)	—	—
Subcutaneous tophi	No	42 (89.36)	39 (100.00)	4.41	0.04*
	Yes	5 (10.64)	0 (0.00)	—	—
Consumption of whole grains	Occasional	24 (48.98)	26 (66.67)	2.77	0.25
	Rarely	23 (46.94)	12 (30.77)	—	—
	Frequently	2 (4.08)	1 (2.56)	—	—
Meat—based soups	Occasionally	21 (42.86)	20 (51.28)	0.77	0.68
	Rarely	6 (12.24)	5 (12.82)	—	—
	Frequently	22 (44.90)	14 (35.90)	—	—
Vegetable soups	Occasionally	16 (33.33)	15 (38.46)	0.26	0.88
	Rarely	3 (6.25)	2 (5.13)	—	—
	Frequently	29 (60.42)	22 (56.41)	—	—
Fresh fruit consumption	Occasionally	24 (48.98)	24 (61.54)	2.17	0.34
	Rarely	12 (24.49)	5 (12.82)	—	—
	Frequently	13 (26.53)	10 (25.64)	—	—
Tea consumption frequency	Occasionally	18 (36.73)	7 (18.42)	3.59	0.17
	Rarely	23 (46.94)	24 (63.16)	—	—
	Frequently	8 (16.33)	7 (18.42)	—	—
Alcohol consumption	Occasionally	19 (38.78)	2 (5.26)	19.01	<0.001*
	Rarely	18 (36.73)	31 (81.58)	—	—
	Frequently	12 (24.49)	5 (13.16)	—	—
Smoking status	No	20 (40.82)	30 (76.92)	11.54	<0.001*
	Yes	29 (59.18)	9 (23.08)	—	—
Exercise frequency	Occasionally	9 (18.37)	14 (35.90)	3.95	0.14
	Rarely	18 (36.73)	9 (23.08)	—	—
	Frequently	22 (44.90)	16 (41.03)	—	—
Age	—	47.57 ± 16.76	51.95 ± 14.14	−1.30	0.20
Height (cm)	—	157.23 ± 7.61	152.64 ± 6.91	2.53	0.01*
Waist circumference (cm)	—	85.81 ± 12.32	74.36 ± 13.30	2.30	0.03*
BMI (kg/m^2^)	—	23.89 ± 3.94	21.21 ± 3.11	2.98	<0.001*

BMI, body mass index; “-” indicates no value; “*” indicates the difference was statistically significant (*p* < 0.05). Values are presented as n (%) or mean ± SD., sample sizes differ between tables due to availability of complete clinical and laboratory data.

Anthropometric measurements also varied significantly. The hyperuricemia/gout group had higher body weights (59.2 vs. 49.4 kg), larger waist circumference (85.8 vs. 74.4 cm), and elevated BMI (23.9 vs. 21.2 kg/m^2^). Height was marginally greater in the affected group (157.23 vs. 152.64 cm, *p* = 0.01). In contrast, no significant group differences were detected in age education level, family history of hyperuricemia and gout, dietary habits, physical activity, sleep patterns, or mobile network usage (Supplementary Table S1). Notably, dietary intakes such as meats, grains, fruits, soup and consumption of processed foods did not differ meaningfully between the groups. Suggesting food choices may be less influential than other factors in this population.

### 3.2 Biochemical indicators in non-hyperuricemia/non-gout and hyperuricemia/gout groups

**FIGURE 1:**
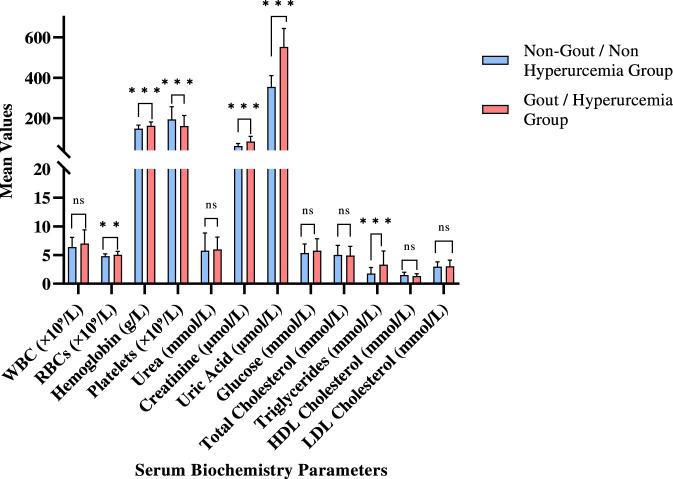
Serum Biochemistry analysis. Comparison between Non-Hyperuricemia/Non-Gout and Hyperuricemia/Gout group. Data are presented as Mean ± SD. Error bars represent standard deviation (SD). “ns” indicates statistically non-significant and “*” indicates statistically significant difference (*, *p* < 0.05; **, *p* < 0.01; ***, *p* < 0.00).

A total of 113 villagers underwent serum biochemistry testing and urinalysis, comprising 56 non-gout/non-hyperuricemia group and 57 gout/hyperuricemia group (hyperuricemia prevalence = 50.44%). As shown in Figure 1 and Supplementary Table S2, several biochemical indices (such as red blood cell (RBC) count, hemoglobin, platelet count, alanine aminotransferase (ALT), total bilirubin, creatinine, uric acid, and triglycerides) differed significantly between groups (*p* < 0.05). Renal function markers showed clearest separation: serum uric acid levels were 56% higher in the hyperuricemia/gout group (553.13 vs. 354.73 μmol/L, *p* < 0.001), and serum creatinine levels were 37% elevated (84.66 vs. 61.80 μmol/L, *p* < 0.001), indicating reduced kidney filtration efficiency.

Hematological parameters also differed. Affected individuals had higher RBC counts (5.08 vs. 4.81 ×10^9^/L, *p* = 0.01) and hemoglobin (162.77 vs. 147.50 g/L, *p* < 0.001), suggesting potential compensatory erythropoiesis or dehydration. Alongside a 17% reduction in platelet count (161.09 vs. 194.14 ×10^9^/L, *p* < 0.001), a finding that may reflect inflammatory consumption or altered marrow activity. Elevated ALT 47% higher in gout patients (28.22 vs. 19.15 U/L, *p* = 0.04) suggested mild hepatic stress. Metabolic differences included higher triglyceride levels (3.35 vs. 1.80 mmol/L, *p* < 0.001), whereas HDL-C was lower but not statistically significant (1.35 vs. 1.52 mmol/L, *p* = 0.06) (Figure 1). White blood cell (WBC) count, total cholesterol, Low-Density Lipoprotein Cholesterol (LDL-C), glucose, and most urinary indicators did not differ significantly. Total bilirubin was unexpectedly lower in the hyperuricemia/gout group (10.50 vs. 13.85 mmol/L, *p* = 0.04), warranting further investigation (Table 3).

**TABLE 3:** Urinalysis parameters among participants with available urine test data, stratified by Non-Hyperuricemia/Non-Gout and Hyperuricemia/Gout status.

Parameters	Subgroup	Hyperuricemia/Gout group (n = 57)	Non-hyperuricemia/Non-gout group (n = 56)	χ^2^/t	*p*-value
Gender	Female	9 (15.79)	41 (73.21)	37.76	<0.001*
	Male	48 (84.21)	15 (26.79)	—	—
Age	**—**	47.63 ± 17.69	48.88 ± 15.72	—	—
Nitrite	Positive	10 (17.54)	12 (21.43)	0.27	0.60
	Negative	47 (82.46)	44 (78.57)	—	—
Vitamin C	Positive	4 (7.02)	5 (8.93)	0.14	0.71
	Negative	53 (92.98)	51 (91.07)	—	—
Occult blood	Positive	3 (5.26)	2 (3.57)	0.19	0.66
	Negative	54 (94.74)	54 (96.43)	—	—
Protein	Positive	1 (1.75)	0 (0.00)	0.99	0.32
	Negative	56 (98.25)	56 (100.00)	—	—
Bilirubin	Positive	5 (8.77)	5 (8.93)	0.00	0.98
	Negative	52 (91.23)	51 (91.07)	—	—
Urobilinogen	Positive	11 (19.30)	11 (19.64)	0.00	0.96
	Negative	46 (80.70)	45 (80.36)	—	—
White blood cells (WBC)	Positive	6 (10.53)	10 (17.86)	1.25	0.26
	Negative	51 (89.47)	46 (82.14)	—	—
pH	—	5.99 ± 0.53	5.94 ± 0.53	0.54	0.59
Specific gravity	—	1.02 ± 0.01	1.02 ± 0.01	−0.68	0.50

“-” indicates no value; “*” indicates the difference was statistically significant (*p* < 0.05). Values are presented as n (%) or mean ± SD., sample sizes differ between tables due to availability of complete clinical and laboratory data.

### 3.3 Urinalysis comparisons

Urinalysis parameters (nitrites, vitamin C, occult blood, protein, urobilinogen, WBC, pH, and specific gravity) showed no statistically significant differences between groups, apart from the expected gender imbalance. These findings suggest that urinary abnormalities were not prominent features distinguishing hyperuricemia/gout from unaffected participants in this population. But the total bilirubin was paradoxically lower in hyperuricemia/gout group (10.50 vs. 13.85 mmol/L, *p* = 0.04), warranting further investigation (Table 3).

### 3.4 Hardy–Weinberg equilibrium test

Eight SNPs in *SLC2A9* (rs3733591, rs16890979, rs7442295, rs2280205, rs10939650, and rs10489070) and *SLC22A1*2 (rs475688 and rs7929627) were analyzed. All SNPs confirmed to Hardy–Weinberg equilibrium (*p* > 0.05; Table 4), supporting reliable genotyping quality and indicating no major deviations due to population structure or selection. Minor allele frequencies were consistent with reference data from the 1,000 Genomes Han Chinese population.

**TABLE 4:** Genetic equilibrium status of *SLC2A9*, and *SLC22A12.*

SNPs		Allele	HWE^a^	MAF	MAF (1,000 g - CHBS)^b^	Inferred LD pair
*SLC2A9*	rs16890979	C/T	1.000	0.013 T	0.010 T	rs7442295 (*r* ^2^ = 1.0)
	rs7442295	A/G	1.000	0.013 G	0.010 G	rs16890979 (*r* ^2^ = 1.0)
	rs10939650	C/T/G	0.704	0.279 C	0.466 C	None
	rs2280205	G/A/C	0.242	0.332 A	0.197 A	Weak LD with rs7929627
	rs3733591	C/T	0.357	0.534 C	0.334 C	Independent SNPs
	rs10489070	C/G	1.000	0.151 G	0.132 G	None
*SLC22A12*	rs7929627	A/G	0.347	0.396 G	0.428 G	Weak LD with rs2280205
	rs475688	C/T	0.401	0.315 T	0.459 T	Low LD (No strong LD candidate)

MAF, minor allele frequency. a, *p* value of the Hardy–Weinberg equilibrium test; b, MAF, of 1000 Genomes Project (Han Chinese in Beijing, China). Although some SNPs, showed deviations in allele frequencies, all were in Hardy-Weinberg equilibrium (HWE, *p* > 0.05), except where noted, and MAF, values were consistent with the reference population.

### 3.5 Linkage disequilibrium (LD) patterns

**FIGURE 2:**
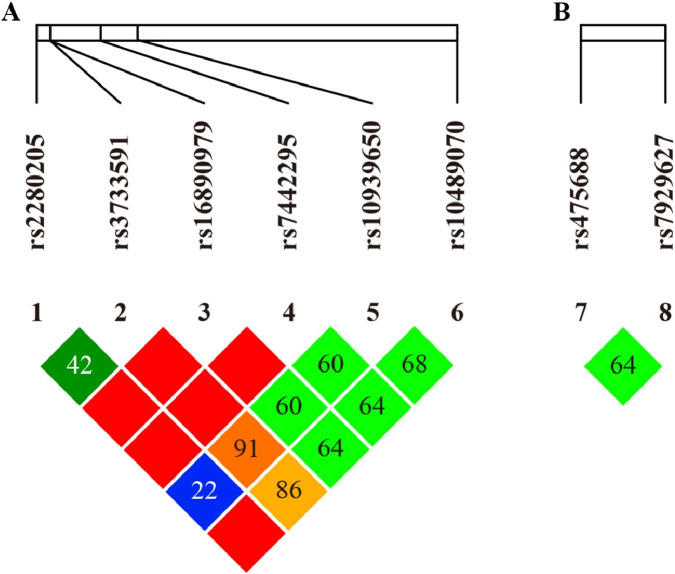
Pairwise linkage disequilibrium (LD) heatmaps. **(A)** *SLC2A9*: rs16890979 and rs7442295 are in complete LD (*r* ^*2*^ = 1.00), forming a single haplotype block while other SNPs pairs show weak to moderate LD. **(B)** *SLC22A12*: overall weak LD was observed among the analyzed SNPs, including rs7929627. Values shown in the diamonds represent *r* ^*2*^ × 100.

Pairwise LD analysis revealed complete LD between *SLC2A9* rs16890979 and rs7442295 (*r* ^*2*^ = 1.00), consistent with their identical minor-allele frequencies (MAF = 0.013) and HWE equilibrium (*p* = 1.00), reveled they are from the same haplotype block (Figure 2A). Other *SLC2A9* SNPs pairs showed weak to moderate LD (all *r* ^*2*^ < 0.8). Within *SLC22A12*, rs7929627 displayed weak LD with *SLC2A9* rs2280205 (Figure 2B), whereas other cross-gene comparisons showed little evidence of LD. No strong LD was observed for rs10489070 or for most remaining SNP pairs (Figure 2; Table 4).

To ensure the quality and reliability of our genotyping results, we evaluated all analyzed SNPs in *SLC2A9* and *SLC22A12* for Hardy-Weinberg Equilibrium (HWE). As shown in Table 4, HWE test confirmed that the observed genotype frequencies for all SNPs, including the gout-associated rs3733591 (p. Arg294His) and rs16890979 (p. Val282Ile) variants in *SLC2A9* as well as the neutral rs475688 in *SLC22A12*, matched expected frequencies under random mating (all *p* > 0.05). This adherence to HWE indicates our genotyping data were technically robust, free from significant population stratification, and unaffected by selection bias. These results provide confidence that the allele frequency differences we detected between gout and non-gout hyperuricemia groups reflect true biological associations rather than technical or population genetic artifacts.

### 3.6 Associations between gene polymorphisms and hyperuricemia/gout

This study examined the associations between genetic variants in the *SLC2A9* and *SLC22A12,* and the risk of hyperuricemia and gout within a Miao population of Longyintan Village. In the first comparison, we analyzed genetic differences between the non-gout/non-hyperuricemia group (MC) and the gout/hyperuricemia group (MGH) within the Miao villagers themselves. No significant difference was observed for the genotype frequencies for any of the eight SNPs studied (Supplementary Table S3). Given the relative isolation of the Longyintan community and the high prevalence of hyperuricemia and gout, we hypothesized a potential genetic predisposition in this population.

To further explore the genetic susceptibility, comparisons were then made between Miao population with gout (MG) and with hyperuricemia (MH) and non-Miao healthy controls (HC) from neighboring villages. These analyses revealed that several *SLC2A9* variants differed significantly between groups, whereas *SLC22A12* variants showed no significant differences.

#### 3.6.1 Genetic associations with gout: MG vs. HC

Table 5 shows that the T allele of *SLC2A9_*rs3733591 was significantly less frequent in the MG compared with HC (61.8% vs. 33.3%, OR = 0.31, 95% CI: 0.10–0.92, *p* = 0.035). In contrast, the T allele of *SLC2A9_*rs10939650 was more frequent (3.95 times higher risk of gout) in MG than HC (83.3% vs. 55.9%, OR = 3.95, 95% CI: 1.11–14.0, *p* = 0.034). Genotype analysis under the codominant model further supported this finding: individuals with TT genotype of rs10939650 had markedly increased gout risk compared to the CC genotype (*p* = 0.009).

**TABLE 5:** Comparison of the allele frequencies between non-Miao villagers from neighboring villages (HC) without Gout/Hyperuricemia group and Miao people from the Longyintan Village with Gout (MG) group.

SNPs	Non-Miao villagers (HC) group		Miao people with gout (MG) group		OR (95% CI)	*p* value
	*n*	%	*n*	%		
***SLC2A9* rs3733591**						
Allele	C: 13	38.2%	16	66.7%	1.000 (ref.)	-
	T: 21	61.8%	8	33.3%	0.31 (0.10–0.92)	0.035*
Codominant model	CC: 3	17.6%	6	50.0%	1.000 (ref.)	-
	CT: 7	41.2%	4	33.3%	0.29 (0.05–1.61)	0.16
	TT: 7	41.2%	2	16.7%	0.14 (0.02–1.04)	0.055
Dominant model	CC: 3	17.6%	6	50.0%	1.000 (ref.)	-
(CT + TT vs. CC)	CT + TT: 14	82.4%	6	50.0%	0.21 (0.04–1.13)	0.069
Recessive model	CC + CT: 10	58.8%	10	83.3%	1.000 (ref.)	-
(TT vs. CC + CT)	TT: 7	41.2%	2	16.7%	0.29 (0.05–1.67)	0.16
***SLC2A9* rs16890979**						
Allele	C: 30	88.2%	24	100	1.000 (ref.)	-
	T: 4	11.8%	0	0%	0.00 (0.00–NA)	0.999
Codominant model	CC: 13	76.5%	12	100%	1.000 (ref.)	-
	CT: 4	23.5%	0	0%	0.00 (0.00–NA)	0.999
	TT: 0	0%	0	0%	NA	NA
Dominant model	CC: 13	76.5%	12	100%	1.000 (ref.)	-
	CT + TT: 4	23.5%	0	0%	0.00 (0.00–NA)	0.999
Recessive model	CC + CT: 17	100%	12	100%	1.000 (ref.)	-
	TT: 0	0%	0	0%	NA	NA
***SLC2A9* rs7442295**						
Allele	A: 30	88.2%	24	100%	NA	NA
	G: 4	11.8%	0	0%	0.00 (0.00–NA)	0.999
Codominant model	AA: 13	76.5%	12	100%	1.000 (ref.)	-
	AG: 4	23.5%	0	0%	0.00 (0.00–NA)	0.999
	GG: 0	0%	0	0%	NA	NA
Dominant model	AA: 13	76.5%	12	100%	1.000 (ref.)	-
	AG + GG: 4	23.5%	0	0%	0.00 (0.00–NA)	0.999
Recessive model	AA + AG: 17	100%	12	100%	1.000 (ref.)	-
	GG: 0	0%	0	0%	NA	NA
***SLC2A9* rs2280205**						
Allele	G: 27	79.4%	16	66.6%	1.000 (ref.)	-
	A: 7	20.6%	8	33.3%	1.93 (0.61–6.09)	0.26
Codominant model	GG: 10	58.8%	6	50.0%	1.000 (ref.)	-
	GA: 7	41.2%	4	33.3%	0.95 (0.20–4.56)	0.95
	AA: 0	0%	2	16.7%	NA	0.11
Dominant model (GA + AA vs. GG)	GG: 10	58.8%	6	50.0%	1.000 (ref.)	-
	GA + AA: 7	41.2%	6	50.0%	1.43 (0.34–5.97)	0.62
Recessive model (AA vs. GG + GA)	GG + GA: 17	100%	10	83.3%	1.000 (ref.)	-
	AA: 0	0%	2	16.7%	NA	0.11
***SLC2A9* rs10939650**						
Allele	C: 15	44.1%	4	16.7%	1.000 (ref.)	-
	T: 19	55.9%	20	83.3%	3.95 (1.11–14.0)	0.034*
Codominant model	CC: 4	23.5%	0	0%	1.000 (ref.)	-
	CT: 7	41.2%	4	33.3%	∞ (0.93– ∞ )	0.055
	TT: 6	35.3%	8	66.7%	∞ (1.79– ∞ )	0.009*
Dominant model	CC: 4	23.5%	0	0%	1.000 (ref.)	-
	CT + TT: 13	76.5%	12	100%	∞ (1.67– ∞ )	0.013*
Recessive model	CC + CT: 11	64.7%	4	33.3%	1.000 (ref.)	-
	TT: 6	35.3%	8	66.7%	3.67 (0.84–15.9)	0.083
***SLC22A12* rs10489070**						
Allele	C: 29	85.3%	20	83.3%	1.000 (ref.)	-
	G: 5	14.7%	4	16.7%	1.16 (0.28–4.77)	0.84
Codominant model	CC: 12	70.6%	8	66.7%	1.000 (ref.)	-
	CG: 5	29.4%	4	33.3%	1.20 (0.25–5.76)	0.82
	GG: 0	0%	0	0%	NA	NA
Dominant model	CC: 12	70.6%	8	66.7%	1.000 (ref.)	-
	CG + GG: 5	29.4%	4	33.3%	1.20 (0.25–5.76)	0.82
Recessive model	CC + CG: 17	100%	12	100%	1.000 (ref.)	-
(GG vs. CC + CG)	GG: 0	0%	0	0%	NA	NA
***SLC22A12* rs7929627**						
Allele	A: 16	47.1%	11	45.8%	1.000 (ref.)	-
	G: 18	52.9%	13	54.2%	1.05 (0.38–2.91)	0.92
Codominant model	AA: 4	23.5%	3	12.5%	1.000 (ref.)	-
	AG: 8	47.1%	5	20.8%	0.83 (0.14–4.97)	0.84
	GG: 5	29.4%	4	16.7%	0.80 (0.12–5.41)	0.82
Dominant model	AA: 4	23.5%	3	12.5%	1.000 (ref.)	-
(AG + GG vs. AA)	AG + GG: 13	76.5%	9	37.5%	0.92 (0.18–4.76)	0.92
Recessive model	AA + AG: 12	70.6%	8	33.3%	1.000 (ref.)	-
	GG: 5	29.4%	4	16.7%	0.60 (0.13–2.83)	0.52
***SLC22A12* rs475688**						
Allele	C: 22	64.7%	20	83.3%	1.000 (ref.)	-
	T: 14	41.2%	4	16.7%	0.37 (0.10–1.32)	0.12
Codominant model	CC: 7	41.2%	8	66.7%	1.000 (ref.)	-
	CT: 8	47.1%	4	33.3%	0.44 (0.09–2.11)	0.30
	TT: 2	11.8%	0	0%	0.00 (0.00–NA)	0.999
Dominant model	CC: 7	41.2%	8	66.7%	1.000 (ref.)	-
	CT + TT: 10	58.8%	4	33.3%	0.35 (0.07–1.67)	0.19
Recessive model	CC + CT: 15	88.2%	12	100%	1.000 (ref.)	-
	TT: 2	11.8%	0	0%	0.00 (0.00–NA)	0.999

OR, odd ratio; CI, confidence interval; “NA” not available; “-” indicates no value; “*” indicates the difference was statistically significant (*p* < 0.05).

Two additional *SLC2A9* variants (rs3733591 and rs10939650) showed nominal associations consistent with these allele-level findings. We suggested that the T allele may have a protective effect against progressing from high uric acid to clinical gout for Miao people. Interestingly, the T allele for rs16890979 and G allele for rs7442295 were completely absent in MG group but were observed in HC group.

#### 3.6.2 Genetic associations with hyperuricemia: MH vs. HC

Hyperuricemia was highly prevalent in the Miao population (>50% among the 114 participants tested, Supplementary Table S2). We therefore compared MH with HC to identify variants associated with elevated uric acid levels (Table 6). Significant differences were found for *SLC2A9* rs16890979, rs7442295, rs2280205 and rs10939650 between HC and MH groups, while the remaining SNPs showed no meaningful differences. For rs2280205 (*SLC2A9*), the A allele was associated with a significantly higher risk (2.48 times higher) of hyperuricemia (OR = 2.48, 95% CI: 1.01–6.12, *p* = 0.048) The association was strongest for the AA genotype under the Recessive Model (*p* = 0.009). Similarly, the T allele of rs10939650 (OR = 2.51, 95% CI: 1.13–5.59, *p* = 0.024) was significantly common in the MH compared with HC (76.1% vs. 55.9%; OR = 2.51, 95% CI: 1.13–5.59, *p* = 0.024). Dominant model analysis was consistent with these results (93.5% vs. 76.5%, OR = 4.41, 95% CI: 1.01–19.3, *p* = 0.049), indicating that both TT and CT genotypes elevate hyperuricemia risk in the Longyintan Village.

**TABLE 6:** Comparison of the allele frequencies between non-Miao villagers from neighboring villages (HC) without Gout/Hyperuricemia group and Miao people from the Longyintan Village with Hyperuricemia (MH) group.

SNPs	Non-Miao villagers (HC) group		Miao people with hyperuricemia (MH) group		OR (95% CI)	p value
	*N*	%	*n*	%		
*SLC2A9* rs3733591						
Allele	C: 13	38.2%	53	57.6%	1.000 (ref.)	-
	T: 21	61.8%	39	42.4%	0.46 (0.21–1.01)	0.053
Codominant model	CC: 3	17.6%	15	32.6%	1.000 (ref.)	-
	CT: 7	41.2%	23	50.0%	0.66 (0.15–2.83)	0.57
	TT: 7	41.2%	8	17.4%	0.23 (0.05–1.10)	0.065
Dominant model	CC: 3	17.6%	15	32.6%	1.000 (ref.)	-
(CT + TT vs. CC)	CT + TT: 14	82.4%	31	67.4%	0.44 (0.11–1.79)	0.25
Recessive model	CC + CT: 10	58.8%	38	82.6%	1.000 (ref.)	-
(TT vs. CC + CT)	TT: 7	41.2%	8	17.4%	0.30 (0.09–1.02)	0.054
*SLC2A9* rs16890979						
Allele	C: 30	88.2%	92	100%	1.000 (ref.)	-
	T: 4	11.8%	0	0%	0.00 (0.00–NA)	<0.001
Codominant model	CC: 13	76.5%	46	100%	1.000 (ref.)	-
	CT: 4	23.5%	0	0%	0.00 (0.00–NA)	0.003*
	TT: 0	0%	0	0%	NA	NA
Dominant model	CC: 13	76.5%	46	100%	1.000 (ref.)	-
	CT + TT: 4	23.5%	0	0%	0.00 (0.00–NA)	0.003*
Recessive model	CC + CT: 17	100%	46	100%	1.000 (ref.)	-
	TT: 0	0%	0	0%	NA	NA
*SLC2A9* rs7442295						
Allele	A: 30	88.2%	92	100%	1.000 (ref.)	-
	G: 4	11.8%	0	0%	0.00 (0.00–NA)	<0.001*
Codominant model	AA: 13	76.5%	46	100%	1.000 (ref.)	-
	AG: 4	23.5%	0	0%	0.00 (0.00–NA)	0.003*
	GG: 0	0%	0	0%	NA	NA
Dominant model	AA: 13	76.5%	46	100%	1.000 (ref.)	-
	AG + GG: 4	23.5%	0	0%	0.00 (0.00–NA)	0.003*
Recessive model	AA + AG: 17	100%	46	100%	1.000 (ref.)	-
	GG: 0	0%	0	0%	NA	NA
*SLC2A9* rs2280205						
Allele	G: 27	79.4%	56	60.9%	1.000 (ref.)	-
	A: 7	20.6%	36	39.1%	2.48 (1.01–6.12)	0.048
Codominant model	GG: 10	58.8%	18	39.1%	1.000 (ref.)	-
	GA: 7	41.2%	20	43.5%	1.59 (0.53–4.74)	0.41
	AA: 0	0%	8	17.4%	∞ (1.67– ∞ )	0.009*
Dominant model (GA + AA vs. GG)	GG: 10	58.8%	18	39.1%	1.000 (ref.)	-
	GA + AA: 7	41.2%	28	60.9%	2.22 (0.78–6.33)	0.14
Recessive model (AA vs. GG + GA)	GG + GA: 17	100%	38	82.6%	1.000 (ref.)	-
	AA: 0	0%	8	17.4%	∞ (1.67– ∞ )	0.009*
*SLC2A9* rs10939650						
Allele	C: 15	44.1%	22	23.9%	1.000 (ref.)	-
	T: 19	55.9%	70	76.1%	2.51 (1.13–5.59)	0.024*
Codominant model	CC: 4	23.5%	3	6.5%	1.000 (ref.)	-
	CT: 7	41.2%	16	34.8%	3.05 (0.62–15.1)	0.17
	TT: 6	35.3%	27	58.7%	6.00 (1.23–29.3)	0.027*
Dominant model	CC: 4	23.5%	3	6.5%	1.000 (ref.)	-
	CT + TT: 13	76.5%	43	93.5%	4.41 (1.01–19.3)	0.049
Recessive model	CC + CT: 11	64.7%	19	41.3%	1.000 (ref.)	-
	TT: 6	35.3%	27	58.7%	2.61 (0.87–7.81)	0.086
*SLC22A12* rs10489070						
Allele	C: 29	85.3%	76	82.6%	1.000 (ref.)	-
	G: 5	14.7%	16	17.4%	1.22 (0.41–3.63)	0.72
Codominant model	CC: 12	70.6%	32	69.6%	1.000 (ref.)	-
	CG: 5	29.4%	12	26.1%	0.90 (0.26–3.09)	0.87
	GG: 0	0%	2	4.3%	∞ (0.29 ∞ )	0.32
Dominant model	CC: 12	70.6%	32	69.6%	1.000 (ref.)	-
	CG + GG: 5	29.4%	14	30.4%	1.05 (0.32–3.46)	0.94
Recessive model	CC + CG: 17	100%	44	95.7%	1.000 (ref.)	-
(GG vs. CC + CG)	GG: 0	0%	2	4.3%	∞ (0.29– ∞ )	0.32
*SLC22A12* rs7929627						
Allele	A: 16	47.1%	58	63.0%	1.000 (ref.)	-
	G: 18	52.9%	34	37%	0.52 (0.24–1.13)	0.099
Codominant model	AA: 4	23.5%	18	39.1%	1.000 (ref.)	-
	AG: 8	47.1%	22	47.8%	0.61 (0.17–2.23)	0.45
	GG: 5	29.4%	6	13.0%	0.27 (0.05–1.36)	0.11
Dominant model	AA: 4	23.5%	18	39.1%	1.000 (ref.)	-
(AG + GG vs. AA)	AG + GG: 13	76.5%	28	60.9%	0.48 (0.14–1.68)	0.25
Recessive model	AA + AG: 12	70.6%	40	87.0%	1.000 (ref.)	-
	GG: 5	29.4%	6	13.0%	0.36 (0.10–1.32)	0.12
*SLC22A12* rs475688						
Allele	C: 22	64.7%	62	67.4%	1.000 (ref.)	-
	T: 14	41.2%	30	32.6%	0.89 (0.40–1.97)	0.77
Codominant model	CC: 7	41.2%	21	45.7%	1.000 (ref.)	-
	CT: 8	47.1%	20	43.5%	0.83 (0.26–2.67)	0.76
	TT: 2	11.8%	5	10.9%	0.83 (0.14–5.06)	0.85
Dominant model	CC: 7	41.2%	21	45.7%	1.000 (ref.)	-
	CT + TT: 10	58.8%	25	54.3%	0.83 (0.28–2.49)	0.74
Recessive model	CC + CT: 15	88.2%	41	89.1%	1.000 (ref.)	-
	TT: 2	11.8%	5	10.9%	0.91 (0.16–5.14)	0.92

OR, odd ratio; CI, confidence interval; “NA” not available; “-” indicates no value; “ ∞ ” indicates infinity which means perfect association of genotype with gout disease; “*” indicates the difference was statistically significant (*p* < 0.05).

As in the gout comparison, the T allele of rs16890979 and G allele of rs7442295 were completely absent in MH group. Overall, results indicate that several *SLC2A9* variants, particularly rs10939650 and rs2280205, may contribute to susceptibility to hyperuricemia and gout in this genetically isolated Miao population. These associations should be interpreted cautiously interpreted cautiously; due to sample size limitations but highlight potential genetic differences between the Miao villagers and neighboring non-Miao groups. Validation in larger, independent cohorts is needed.

## 4 Discussion

This cross-sectional study examined lifestyle, biochemical, and genetic factors associated with hyperuricemia and gout prevalence in Longyintan Village, a relatively isolated Miao community in Yunnan Province, China. By comparing individuals with and without hyperuricemia/gout, and further contrasting Miao participants with non-Miao villagers, we sought to clarify both environmental and inherited contributors to disease susceptibility. Eight widely studied SNPs *SLC2A9* and *SLC22A12*, two key urate transport genes, were evaluated for their potential role in this population.

Consistent with previous work, our results showed a marked sex difference. The prevalence of hyperuricemia and gout was substantially higher in men than women (83.67% in males vs. 28.21% in females, *p* < 0.001). This aligns with earlier observations that men are more prone to hyperuricemia due to lower renal urate clearance and the protective influence of estrogen in women (Eun et al., 2021; Jaiprabhu et al., 2025; Lee et al., 2024; van Veen and Haeri, 2015). With the advancement of social and economic conditions, the number of obese people is increasing rapidly. Many studies found that obesity was an important risk factor for hyperuricemia and gout (Cao et al., 2017; Evans et al., 2018; Zhang et al., 2025). Obesity-related measures, including body weight (59.20 vs. 49.43 kg, *p* < 0.001), waist circumference (85.81 vs. 74.36 cm, *p* = 0.03), and BMI (23.89 vs. 21.21 kg/m^2^, *p* < 0.001) were significantly higher among affected individuals, further supporting evidence that adiposity promotes elevated uric acid levels through insulin resistance and impaired renal urate elimination (Cao et al., 2017; Dong et al., 2020; Evans et al., 2018; Zhang et al., 2025).

In contrast to expectations and previous reports that low-purine diets (fruits, eggs, legumes, dairy products, and whole grains) may reduce serum uric acid levels key factors in gout management (Aihemaitijiang et al., 2020; Zhang et al., 2022b; Chen et al., 2024). Our analysis found no significant dietary differences (e.g., meats and seafood, all *p* > 0.05) between the hyperuricemia/gout and non-hyperuricemia/non-gout groups of Miao people from the Longyintan Village. Lifestyle behaviors, however, showed clear associations: smoking (59.18% vs. 23.08%, *p* < 0.001) and frequent alcohol consumption (24.49% vs. 13.16%, *p* < 0.001) were substantially more common among individuals with hyperuricemia/gout, consistent with the established role of these exposures in increasing urate production and impairing renal excretion (Neogi et al., 2014; Yang et al., 2017).

The biochemical results further emphasized the metabolic burden (association of hyperuricemia and gout to renal impairment) on an affected individual. Elevated serum triglyceride (3.35 ± 2.35 mmol/L vs. 1.80 mmol/L, *p* < 0.001) and lower HDL-C (1.35 vs. 1.52 mmol/L, *p* = 0.06) reflect dyslipidemia commonly observed in hyperuricemia, reinforcing the link between urate metabolism and metabolic syndrome and can be a potential marker to predict the gout at an early stage (Lu et al., 2020; Luo et al., 2022; Xian et al., 2025). Hematological differences, including elevated RBC count (5.08 vs. 4.81 ×10^9^/L, *p* = 0.01) and hemoglobin (162.77 vs. 147.50 g/L, *p* < 0.001) alongside reduced platelets (161.09 vs. 194.14 ×10^9^/L, *p* < 0.001), may reflect chronic low-grade inflammation or metabolic alterations associated with hyperuricemia and gout (Yang et al., 2022). Elevated ALT (28.22 vs. 19.15 U/L, *p* = 0.04) and AST (39.63 vs. 28.42 U/L, *p* = 0.06) also suggest hepatic stress, in line with prior studies linking urate dysregulation to liver dysfunction (Thottam et al., 2017; Li et al., 2024a). Our observation of elevated liver enzymes is consistent with other studies reporting systemic metabolic involvement in gout and hyperuricemia. Although several biochemical parameters showed statistically significant differences between groups, the magnitude of some effects was modest. Therefore, these findings should be interpreted cautiously, as statistical significance does not always equate to clinical relevance, and validation in larger, multi-center cohorts is warranted.

Genetic analyses highlighted notable patterns in *SLC2A9.* Strong LD was observed between rs16890979 and rs7442295 (*r* ^2^ = 1.0) (Figure 2) because of identical minor allele frequencies (MAF = 0.013) and HWE equilibrium (*p* = 1.00), consistent with previous findings in Chinese and other East Asian populations (Yang et al., 2014; Merriman, 2015; Yang et al., 2018). Although rs3733591 showed only moderate LD, it its T allele demonstrated a protective association against gout in the Miao ethnic group (OR = 0.31, *p* = 0.035, Table 5). This contrasts with its association with hyperuricemia in Han Chinese (Lin et al., 2024), suggesting ancestry-dependent allele effects, as reported in multiethnic studies (Tu et al., 2010). The complete absence of rs16890979-T and rs7442295-G minor alleles in Miao gout cases (*p* < 0.001, Table 6), may reflect a protective founder effect or selection pressures unique to this isolated population; these patterns differ substantially from European cohorts, where these variants showed weaker or inconsistent associations (Köttgen et al., 2013). The rs10939650-T allele (*SLC2A9*) exhibited a strong risk effect for both gout (OR = 3.95, *p* = 0.034, Table 5), and hyperuricemia (OR = 2.51, *p* = 0.024). This trend corresponds with prior evidence suggesting reduced GLUT9 transporter activity associated with this allele (Wu et al., 2024). Together, these findings indicate a prominent role for *SLC2A9* in urate handling across populations, although the strength and direction of associations may vary by ancestry vary significantly across ancestries (Flynn et al., 2013; Misawa et al., 2020) and it showed the ethnic-specific genetic architecture of *SLC2A9* gene.

In contrast, *SLC22A12* polymorphisms (e.g., rs10489070, rs475688, and rs7929627) did not show significant associations in this study (*p* > 0.05, Tables 5 and 6), consistent with some reports in Chinese and multiethnic cohorts (Shima et al., 2006; Matsuo et al., 2016; Sandoval-Plata et al., 2021). Although rs475688 has been associated with gout in Japanese and in European and Japanese studies (Shima et al., 2006; Matsuo et al., 2016; Sandoval-Plata et al., 2021; Toyoda et al., 2021), our results align with evidence that *SLC22A12* variants like rs121907892 effects are highly population-specific and may be weaker or absent in certain ethnic groups, including the Miao (Flynn et al., 2013; Misawa et al., 2020; Ruiz et al., 2018). The protective effect of rs3733591-T in the Miao group, together with the increased risk associated with the rs10939650-T alleles, further supports the central involvement of *SLC2A9* in urate transport dysregulation (Liu et al., 2022; Wu et al., 2023a; Wu et al., 2024). These variants, combined with lifestyle and metabolic factors, may contribute to the elevated hyperuricemia and gout burden observed in the Longyintan populations, as reflected in the stratified analyses (Table 6). In contrast, the neutrality of *SLC22A12* SNPs in our cohort reinforces the gene’s ancestry-dependent penetrance, necessitating caution in trans-ethnic genetic risk modeling (Flynn et al., 2013; Misawa et al., 2020). Although rs10939650 and rs3733591 showed a nominal association (uncorrected *p* = 0.034 and *p* = 0.035, respectively), rs10939650 did not reach the Bonferroni-adjusted threshold (*p* < 0.00625). This indicates that the observed associations should be interpreted as preliminary and require validation in larger cohorts. Besides, our results suggest these alleles may be strongly protective against developing high uric acid levels in this population. The odds ratio (OR) of 0.00 and highly significant *p*-values (<0.001, 0.003) indicate a perfect or near-perfect negative association. The fact that these alleles are present in controls but absent in cases is a very striking finding. Based on these findings there is a need for replication of larger cohorts.

In this study, small sample sizes, particularly for rare variants such as rs2280205-A, limit the statistical power to detect modest genetic effects and hinder the meaningful functional interpretation of these SNPs. The absence of significance after Bonferroni correction further indicates that these findings should be considered preliminary. Several *SLC2A9* variants (rs10939650, rs2280205, and rs3733591) showed suggestive associations with hyperuricemia and gout in the isolated Miao population, whereas *SLC22A12* variants showed no significant effects. Larger, well-powered studies are needed to validate these associations and to explore interactions between *SLC2A9* variants, dietary factors and comorbidities in the wider Yunnan population. These results emphasize the role of population-specific genetic architecture in urate-related disorders and the need for future studies incorporating larger cohorts and functional analyses to clarify gene–environment interactions involved in urate metabolism.

## Data Availability

The datasets presented in this study can be found in online repositories. The names of the repository/repositories and accession number(s) can be found in the article/Supplementary Material.
