# Supplementary material for: Preliminary analysis of lifestyle and genetic factors for hyperuricemia and gout prevalence in the Yunnan Miao population of China
Source: Front Genet. 2026 Jan 29;17:1729712. doi: 10.3389/fgene.2026.1729712 (PMC12893670; doi:10.3389/fgene.2026.1729712)
Supplement: Supplementary file 1 [file Table1.docx]

Supplementary Material

**Table S1.** Demographic and Clinical Comparisons Between Non-Gout / Non-Hyperuricemia and Gout / Hyperuricemia Groups.

| **Category** | **Subgroup** | **Hyperuricemia / Gout Group (n = 49)** | **Non-Hyperuricemia / Non-Gout Group (n = 39)** | **χ² / t Value** | ***p* Value** |
| --- | --- | --- | --- | --- | --- |
| **Gender** | Female | 8(16.33) | 28(71.79) | 27.64 | <0.001^*^ |
|  | Male | 41(83.67) | 11(28.21) | - | - |
| **Family history of hyperuricemia** | No | 39(79.59) | 36(92.31) | 2.79 | 0.09 |
|  | Yes | 10(20.41) | 3(7.69) | - | - |
| **Family history of Gout** | No | 39(79.59) | 35(89.74) | 1.67 | 0.20 |
|  | Yes | 10(20.41) | 4(10.26) | - | - |
| **Medical history** | None | 29(60.42) | 35(89.74) | 9.52 | <0.001* |
|  | Present | 19(39.58) | 4(10.26) | - | - |
| **Non-Traumatic Joint Pain** | No | 35(71.43) | 37(94.87) | 8.02 | <0.001* |
|  | Yes | 14(28.57) | 2(5.13) | - | - |
| **Subcutaneous Tophi** | No | 42(89.36) | 39(100.00) | 4.41 | 0.04* |
|  | Yes | 5(10.64) | 0(0.00) | - | - |
| **Consumption of Whole Grains** | Occasional | 24(48.98) | 26(66.67) | 2.77 | 0.25 |
|  | Rarely | 23(46.94) | 12(30.77) | - | - |
|  | Frequently | 2(4.08) | 1(2.56) | - | - |
| **Meat-based soups** | Occasionally | 21(42.86) | 20(51.28) | 0.77 | 0.68 |
|  | Rarely | 6(12.24) | 5(12.82) | - | - |
|  | Frequently | 22(44.90) | 14(35.90) | - | - |
| **Vegetable soups** | Occasionally | 16(33.33) | 15(38.46) | 0.26 | 0.88 |
|  | Rarely | 3(6.25) | 2(5.13) | - | - |
|  | Frequently | 29(60.42) | 22(56.41) | - | - |
| **Fresh fruit consumption** | Occasionally | 24(48.98) | 24(61.54) | 2.17 | 0.34 |
|  | Rarely | 12(24.49) | 5(12.82) | - | - |
|  | Frequently | 13(26.53) | 10(25.64) | - | - |
| **Tea consumption frequency** | Occasionally | 18(36.73) | 7(18.42) | 3.59 | 0.17 |
|  | Rarely | 23(46.94) | 24(63.16) | - | - |
|  | Frequently | 8(16.33) | 7(18.42) | - | - |
| **Alcohol consumption** | Occasionally | 19(38.78) | 2(5.26) | 19.01 | <0.001* |
|  | Rarely | 18(36.73) | 31(81.58) | - | - |
|  | Frequently | 12(24.49) | 5(13.16) | - | - |
| **Smoking status** | No | 20(40.82) | 30(76.92) | 11.54 | <0.001* |
|  | Yes | 29(59.18) | 9(23.08) | - | - |
| **Exercise frequency** | Occasionally | 9(18.37) | 14(35.90) | 3.95 | 0.14 |
|  | Rarely | 18(36.73) | 9(23.08) | - | - |
|  | Frequently | 22(44.90) | 16(41.03) | - | - |
| **Age** | - | 47.57±16.76 | 51.95±14.14 | -1.30 | 0.20 |
| **Height (cm)** | - | 157.23±7.61 | 152.64±6.91 | 2.53 | 0.01* |
| **Weight (kg)** | - | 59.20±11.44 | 49.43±8.67 | 3.80 | <0.001* |
| **Waist Circumference (cm)** | - | 85.81±12.32 | 74.36±13.30 | 2.30 | 0.03* |
| **BMI (kg/m^2^)** | - | 23.89±3.94 | 21.21±3.11 | 2.98 | <0.001* |
| **Education** | Middle School | 13(27.08) | 4(10.26) | 4.90 | 0.09 |
|  | Below Middle School | 34(70.83) | 35(89.74) | - | - |
|  | High School/College | 1(2.08) | 0(0.00) | - | - |
| **Staple food types** | Both | 12(24.49) | 10(25.64) | 0.02 | 0.90 |
|  | Mainly rice and its products | 37(75.51) | 29(74.36) | - | - |
| **Types of Dishes** | Mixed (Both) | 35(71.43) | 31(79.49) | 0.92 | 0.63 |
|  | Meat-based | 1(2.04) | 1(2.56) | - | - |
| **Egg and egg products** | Occasionally | 35(71.43) | 30(76.92) | 0.40 | 0.82 |
|  | Rarely | 7(14.29) | 4(10.26) | - | - |
|  | Frequently | 7(14.29) | 5(12.82) | - | - |
| **Animal organ meats** | Occasionally | 30(61.22) | 21(53.85) | 0.90 | 0.64 |
|  | Rarely | 13(26.53) | 14(35.90) | - | - |
|  | Frequently | 6(12.24) | 4(10.26) | - | - |
| **Prepared/processed foods** | Occasionally | 17(34.69) | 7(17.95) | 3.72 | 0.16 |
|  | Rarely | 9(18.37) | 12(30.77) | - | - |
|  | Frequently | 23(46.94) | 20(51.28) | - | - |
| **Legumes and bean products** | Occasionally | 35(71.43) | 27(69.23) | 0.67 | 0.71 |
|  | Rarely | 6(12.24) | 7(17.95) | - | - |
|  | Frequently | 8(16.33) | 5(12.82) | - | - |
| **Fried foods** | Occasionally | 30(61.22) | 18(46.15) | 2.83 | 0.24 |
|  | Rarely | 13(26.53) | 17(43.59) | - | - |
|  | Frequently | 6(12.24) | 4(10.26) | - | - |
| **Dairy products** | Occasionally | 23(46.94) | 21(53.85) | 0.87 | 0.65 |
|  | Rarely | 23(46.94) | 17(43.59) | - | - |
|  | Frequently | 3(6.12) | 1(2.56) | - | - |
| **Consumption of mushrooms and fungi** | Occasionally | 39(79.59) | 29(74.36) | 2.59 | 0.27 |
|  | Rarely | 8(16.33) | 10(25.64) | - | - |
|  | Frequently | 2(4.08) | 0(0.00) | - | - |
| **Consumption of pickled/preserved vegetables** | Occasionally | 38(77.55) | 32(82.05) | 3.42 | 0.18 |
|  | Rarely | 7(14.29) | 7(17.95) | - | - |
|  | Frequently | 4(8.16) | 0(0.00) | - | - |
| **Consumption of freshwater products (fish/shellfish)** | Occasionally | 34(69.39) | 28(71.79) | 0.17 | 0.92 |
|  | Rarely | 13(26.53) | 10(25.64) | - | - |
|  | Frequently | 2(4.08) | 1(2.56) | - | - |
| **Consumption of seafood (marine products)** | Occasionally | 15(30.61) | 15(38.46) | 2.79 | 0.25 |
|  | Rarely | 31(63.27) | 24(61.54) | - | - |
|  | Frequently | 3(6.12) | 0(0.00) | - | - |
| **Hot pot consumption** | Occasionally | 32(65.31) | 26(66.67) | 4.58 | 0.10 |
|  | Rarely | 12(24.49) | 13(33.33) | - | - |
|  | Frequently | 5(10.20) | 0(0.00) | - | - |
| **Barbecue/grilled food consumption** | Occasionally | 33(67.35) | 24(61.54) | 3.47 | 0.18 |
|  | Rarely | 13(26.53) | 15(38.46) | - | - |
|  | Frequently | 3(6.12) | 0(0.00) | - | - |
| **Daily water intake** | 1000-2000ml | 28(57.14) | 16(41.03) | 4.64 | 0.10 |
|  | Below 1000ml | 17(34.69) | 22(56.41) | - | - |
|  | Above 2000ml | 4(8.16) | 1(2.56) | - | - |
| **Tea consumption frequency** | Occasionally | 18(36.73) | 7(18.42) | 3.59 | 0.17 |
|  | Rarely | 23(46.94) | 24(63.16) | - | - |
|  | Frequently | 8(16.33) | 7(18.42) | - | - |
| **High-fructose beverage intake** | Occasionally | 23(46.94) | 23(60.53) | 1.59 | 0.45 |
|  | Rarely | 19(38.78) | 11(28.95) | - | - |
|  | Frequently | 7(14.29) | 4(10.53) | - | - |
| **Daily work and rest schedule regular** | Irregular | 4(8.16) | 2(5.13) | 4.85 | 0.09 |
|  | Moderately regular | 15(30.61) | 21(53.85) | - | - |
|  | Regular | 30(61.22) | 16(41.03) | - | - |
| **Bedtime** | 21-23 PM | 37(75.51) | 32(82.05) | 0.95 | 0.62 |
|  | Before 21 | 5(10.20) | 4(10.26) | - | - |
|  | After 23 | 7(14.29) | 3(7.69) | - | - |
| **Wake-up time** | 5-7 AM | 16(32.65) | 15(38.46) | 1.05 | 0.59 |
|  | Before 5 AM | 1(2.04) | 0(0.00) | - | - |
|  | After 7 | 32(65.31) | 24(61.54) | - | - |
| **Type of Mobile network Usage** | None | 2(4.08) | 3(7.69) | 4.27 | 0.23 |
|  | China Telecom | 11(22.45) | 3(7.69) | - | - |
|  | China Mobile | 35(71.43) | 31(79.49) | - | - |
|  | China Unicom | 1(2.04) | 2(5.13) | - | - |
| **Induction cooker/microwave use** | Occasionally | 6(12.24) | 5(12.82) | 1.92 | 0.38 |
|  | Rarely | 5(10.20) | 8(20.51) | - | - |
|  | Frequently | 38(77.55) | 26(66.67) | - | - |

**Notes**: BMI, body mass index; “-” indicates no value; “*” indicates the difference was statistically significant (*p* < 0.05). Values are presented as n (%) or mean ± SD. Sample sizes differ between tables due to availability of complete clinical and laboratory data.

**Table S2.** Biochemical comparison Between Non-Hyperuricemia / Non-Gout and Hyperuricemia / Gout Groups.

| **Parameters** | **Hyperuricemia / Gout Group (n = 57)** | **Non-Hyperuricemia / Non-Gout Group (n = 56)** | **χ² / t** | ***p*-value** |
| --- | --- | --- | --- | --- |
| **WBC (×10⁹/L)** | 7.02±2.38 | 6.41±1.66 | 1.58 | 0.12 |
| **Red Blood Cells (RBCs) (×10⁹/L)** | 5.08±0.57 | 4.81±0.41 | 2.86 | 0.01* |
| **Hemoglobin (g/L)** | 162.77±17. | 147.50±17.59 | 0.19 | 0.66 |
| **Platelets (×10⁹/L)** | 161.09±52.63 | 194.14±62.71 | -3.04 | <0.001* |
| **Alanine Aminotransferase (ALT) (U/L)** | 28.22±11.08 | 19.15±8.37 | 2.17 | 0.04* |
| **Aspartate Aminotransferase (AST) (U/L)** | 39.63±16.67 | 28.42±7.86 | 2.02 | 0.06 |
| **Total Bilirubin (mmol/L)** | 10.50±3.12 | 13.85±3.56 | -2.24 | 0.04* |
| **Urea (mmol/L)** | 5.99±2.14 | 5.80±3.04 | 0.38 | 0.70 |
| **Creatinine (μmol/L)** | 84.66±24.95 | 61.80±12.18 | 6.20 | <0.001* |
| **Uric Acid (μmol/L)** | 553.13±90.98 | 354.73±56.28 | 13.97 | <0.001* |
| **Glucose (mmol/L)** | 5.77±2.06 | 5.35±1.58 | 1.21 | 0.23 |
| **Total Cholesterol (mmol/L)** | 4.93±1.56 | 5.01±1.68 | -0.26 | 0.80 |
| **Triglycerides (mmol/L)** | 3.35±2.35 | 1.80±1.02 | 4.57 | <0.001* |
| **High-Density Lipoprotein (HDL) Cholesterol (mmol/L)** | 1.35±0.41 | 1.52±0.49 | -1.92 | 0.06 |
| **Low-Density Lipoprotein (LDL) Cholesterol (mmol/L)** | 3.03±1.10 | 2.99±0.81 | 0.22 | 0.82 |

**Note**: “*” indicates the difference was statistically significant (*p* < 0.05). Values are presented as mean ± SD. Red blood cell counts are expressed in ×10⁹/L according to the reporting units of the clinical laboratory.

**Table S3:** Comparison of the allele frequencies between groups Miao group (MG) vs. Miao control group (MC).

| **SNPs** | **Miao Gout group (MG)** | | | **Miao control group (MC)** | | **OR (95% CI)** | ***p* value** |
| --- | --- | --- | --- | --- | --- | --- | --- |
|  | *n* | % | | *n* | % |  |  |
| ***SLC2A9*_rs3733591** | | | | | | | |
| Allele | C: 16 | | 66.7% | 66 | 57.9% | 1.000 (ref.) | - |
|  | T: | | 33.3% | 48 | 42.1% | **0.68 (0.28–1.66)** | 0.400 |
| Codominant model | CC: 6 | | 50.0% | 20 | 35.1% | 1.000 (ref.) | - |
|  | CT: 4 | | 33.3% | 26 | 45.6% | **0.51 (0.13–2.04)** | 0.340 |
|  | TT: 2 | | 16.7% | 11 | 19.3% | **0.61 (0.11–3.45)** | 0.570 |
| Dominant model | CC: 6 | | 50.0% | 20 | 35.1% | 1.000 (ref.) | - |
| (CT + TT vs. CC) | CT+TT: 6 | | 50.0% | 37 | 64.9% | **0.51 (0.15–1.71)** | 0.270 |
| Recessive model | CC + CT: 10 | | 83.3% | 46 | 80.7% | 1.000 (ref.) | - |
| (TT vs. CC + CT) | TT: 2 | | 16.7% | 11 | 19.3% | **0.84 (0.17–4.19)** | 0.830 |
| ***SLC2A9*_rs16890979** | | | | | | | |
| Allele | C:24 | | 100% | 114 | 100% | Undefined | 1.000 |
|  | T: 0 | | 0% | 0 | 0% | Undefined | **-** |
| Codominant model | CC: 12 | | 100% | 57 | 100% | Undefined | 1.000 |
|  | CT: 0 | | 0% | 0 | 0% | Undefined | **-** |
|  | TT: 0 | | 0% | 0 | 0% | Undefined | **-** |
| Dominant model | CC: 12 | | 100% | 57 | 100% | Undefined | 1.000 |
|  | CT+TT: 0 | | 0% | 0 | 0% | Undefined | **-** |
| Recessive model | CC+CT: 12 | | 100% | 57 | 100% | Undefined | 1.000 |
|  | TT: 0 | | 0% | 0 | 0% | Undefined | **-** |
| ***SLC2A9*_rs7442295** | | | | | | | |
| Allele | A:24 | | 100% | 114 | 100% | Undefined | 1.000 |
|  | G:0 | | 0% | 0 | 0% | Undefined | - |
| Codominant model | AA: 12 | | 100% | 57 | 100% | Undefined | 1.000 |
|  | AG: 0 | | 0% | 0 | 0% | Undefined | - |
|  | GG: 0 | | 0% | 0 | 0% | Undefined | - |
| Dominant model | AA: 12 | | 100% | 57 | 100% | Undefined | 1.000 |
|  | AG+GG: 0 | | 0% | 0 | 0% | Undefined | - |
| Recessive model | AA+AG: 12 | | 100% | 57 | 100% | Undefined | 1.000 |
|  | GG: 0 | | 0% | 0 | 0% | Undefined | **-** |
| ***SLC2A9*_rs2280205** | | | | | | | |
| Allele | G: 16 | | 66.7% | 74 | 64.9% | 1.000 (ref.) | - |
|  | A: 8 | | 33.3% | 40 | 35.1% | **0.92 (0.38–2.25)** | 0.860 |
| Codominant model | GG: 6 | | 50.0% | 24 | 42.1% | 1.000 (ref.) | - |
|  | GA: 4 | | 33.3% | 26 | 45.6% | **0.62 (0.16–2.36)** | 0.480 |
|  | AA: 2 | | 16.7% | 7 | 12.3% | **1.14 (0.20–6.58)** | 0.880 |
| Dominant model  (GA + AA vs. GG) | GG: 6 | | 50.0% | 24 | 42.1% | 1.000 (ref.) | - |
|  | GA+AA: 6 | | 50.0% | 33 | 57.9% | **0.73 (0.22–2.42)** | 0.600 |
| Recessive model  (AA vs. GG + GA) | GG+GA: 10 | | 83.3% | 50 | 87.7% | 1.000 (ref.) | - |
|  | AA: 2 | | 16.7% | 7 | 12.3% | **1.43 (0.26–7.80)** | 0.680 |
| ***SLC2A9*_rs10939650** | | | | | | | |
| Allele | C: 4 | | 16.7% | 31 | 27.2% | 1.000 (ref.) | - |
|  | T: 20 | | 83.3% | 83 | 72.81% | **1.86 (0.59–5.89)** | 0.290 |
| Codominant model | CC: 0 | | 0% | 4 | 7.0% | Undefined | 1.000 |
|  | CT: 4 | | 33.3% | 23 | 40.4% | **1.38 (0.13–14.7)** | 1.000 |
|  | TT: 8 | | 66.7% | 30 | 52.6% | **2.13 (0.20–22.4)** | 0.530 |
| Dominant model | CC: 0 | | 0% | 4 | 7.0% | 1.000 (ref.) | - |
|  | CT + TT: 12 | | 100% | 53 | 93.0% | Undefined | 1.000 |
| Recessive model | CC + CT: 4 | | 33.3% | 27 | 47.4% | 1.000 (ref.) | - |
|  | TT: 8 | | 66.7% | 30 | 52.6% | **1.80 (0.50–6.50)** | 0.370 |
| ***SLC22A12*_rs10489070** | | | | | | | |
| Allele | C: 20 | | 83.3% | 100 | 87.7% | 1.000 (ref.) | - |
|  | G: 4 | | 16.7% | 14 | 12.3% | **1.43 (0.42–4.83)** | 0.570 |
| Codominant model | CC: 8 | | 66.7% | 43 | 75.4% | 1.000 (ref.) | - |
|  | CG: 4 | | 33.3% | 14 | 24.6% | **1.54 (0.40–5.89)** | 0.530 |
|  | GG: 0 | | 0% | 0 | 0% | Undefined | 1.000 |
| Dominant model | CC: 8 | | 66.7% | 43 | 75.4% | 1.000 (ref.) | - |
|  | CG + GG: 4 | | 33.3% | 14 | 24.6% | **1.54 (0.40–5.89)** | 0.530 |
| Recessive model | CC + CG: 12 | | 100% | 57 | 100% | Undefined | 1.000 |
| (GG vs. CC + CG) | GG: 0 | | 0% | 0 | 0% | Undefined | 1.000 |
| ***SLC22A12*_rs7929627** | | | | | | | |
| Allele | A: 11 | | 45.8% | 71 | 61.2% | 1.000 (ref.) | - |
|  | G: 13 | | 54.2 % | 43 | 37.7 % | **1.95 (0.83–4.59)** | 0.126 |
| Codominant model | AA: 2 | | 16.7% | 22 | 37.9% | 1.000 (ref.) | - |
|  | AG: 7 | | 58.3% | 27 | 46.6% | **2.85 (0.55–14.8)** | 0.211 |
|  | GG: 3 | | 25.0% | 8 | 13.8% | **4.13 (0.62–27.5)** | 0.143 |
| Dominant model | AA: 2 | | 16.7% | 22 | 37.9% | 1.000 (ref.) | - |
| (AG + GG vs. AA) | AG + GG: 10 | | 83.3% | 35 | 60.3% | **3.14 (0.65–15.2)** | 0.156 |
| Recessive model | AA + AG: 9 | | 75.0% | 49 | 84.5% | 1.000 (ref.) | - |
|  | GG: 3 | | 25.0% | 8 | 13.8% | **2.04 (0.47–8.86)** | 0.344 |
| ***SLC22A12*_rs475688** | | | | | | | |
| Allele | C:20 | | 83.3% | 90 | 79% | 1.000 (ref.) | - |
|  | T: 4 | | 16.7% | 34 | 29.8% | **0.75 (0.23–2.42)** | 0.630 |
| Codominant model | CC: 8 | | 66.7% | 36 | 63.2% | 1.000 (ref.) | - |
|  | CT: 4 | | 33.3% | 18 | 31.6% | **1.00 (0.27–3.72)** | 1.000 |
|  | TT: 0 | | 0% | 3 | 5.3% | Undefined | 1.000 |
| Dominant model | CC: 8 | | 66.7% | 36 | 63.2% | 1.000 (ref.) | - |
|  | CT + TT: 4 | | 33.3% | 21 | 36.8% | **0.86 (0.24–3.07)** | 0.810 |
| Recessive model | CC + CT: 12 | | 100% | 54 | 94.7% | 1.000 (ref.) | - |
|  | TT: 0 | | 0% | 3 | 5.3% | Undefined | 1.000 |

**Notes:** OR, Odd Ratio; CI, Confidence interval; “-” indicates no value; “*” indicates the difference was statistically significant (*p* < 0.05).

**
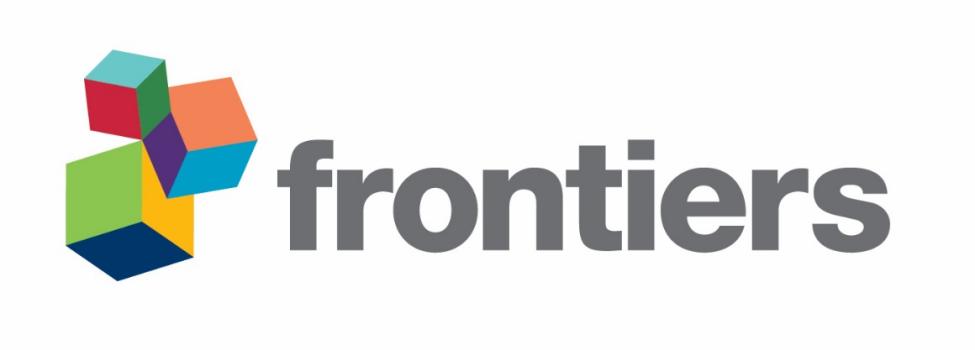
**
